# Artificial intelligence and the cardiologist: what you need to know for 2020

**DOI:** 10.1136/heartjnl-2019-316033

**Published:** 2020-01-23

**Authors:** Antonio de Marvao, Timothy JW Dawes, James Philip Howard, Declan P O'Regan

**Affiliations:** 1 MRC London Institute of Medical Sciences, Imperial College London, London, UK; 2 National Heart and Lung Institute, Imperial College London, London, UK

We live in an era with unprecedented availability of clinical and biological data that include electronic health records, wearable sensors, biomedical imaging and multiomics. The scale, complexity and rate at which such data are collected require innovative approaches to statistics and computer science that draw on the rapid advances in artificial intelligence (AI) for efficiently identifying actionable insights into disease processes. A basic understanding of AI’s strengths, applications and limitations is now essential for researchers and clinical cardiologists.

**Figure 1:**
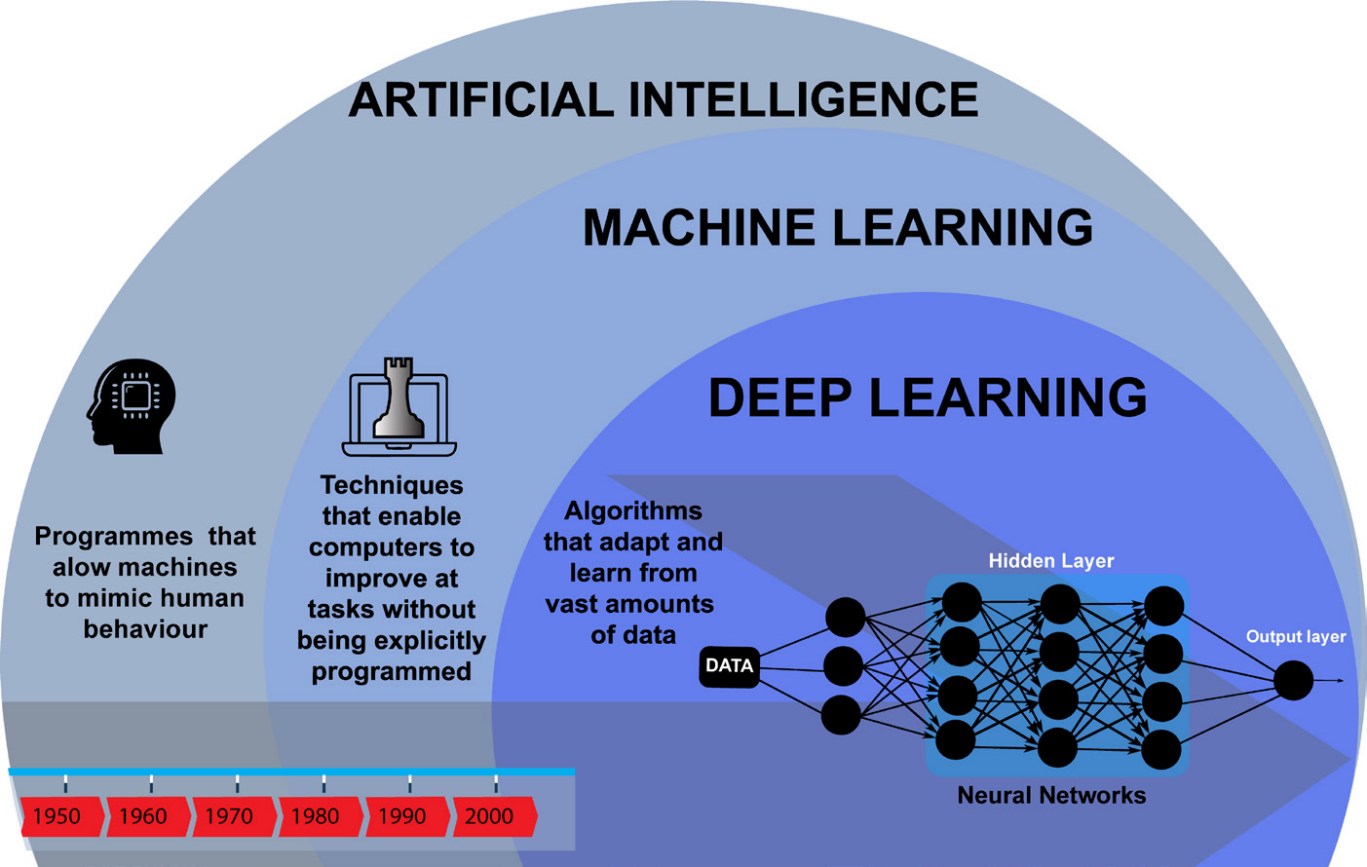
Artificial Intelligence through time.

In this context, AI refers to a collection of computational concepts that can be summarised as a machine’s ability to generalise learning in order to efficiently achieve complex tasks autonomously. Machine learning (ML) achieves this by using algorithms to improve task performance without needing to be explicitly programmed and can be broadly divided into supervised and unsupervised approaches. In supervised learning, the mapping between paired input and output variables is iteratively optimised for use in regression and classification tasks. In unsupervised learning, only input data are available and algorithms are used to find inherent clusters or associations. In recent years, ML has become dominated by deep learning (DL), which is a methodology using multilayer neural networks to progressively obtain more abstract representations of complex data. Figure 1 provides a high-level schematic of the field of AI.

A DL algorithm consists of three types of layer: an input layer, hidden layers and an output layer. The architecture of the network and initial training variables (hyperparameters) are predetermined. Each neuron has an activation function that defines an output from a given set of weighted inputs from other neurons. During training, we measure how wrong the model is, using a cost function, and improve the algorithm’s performance by incrementally optimising the weights between neurons using a gradient descent function. An important class of DL in medical image recognition is convolutional neural networks in which smaller generic spatial features are abstracted from the input data. The many iterations of training, as well as hyperparameter optimisation, often require the parallel processing capabilities of a high-performance graphics processing unit (GPU), although inference often requires much less processing power. An unbiased estimate of how well an algorithm generalises may be obtained by partitioning the available data using internal validation, for example, nested cross validation or bootstrapping, or preferably by external validation using an unseen independent dataset.

**Figure 2:**
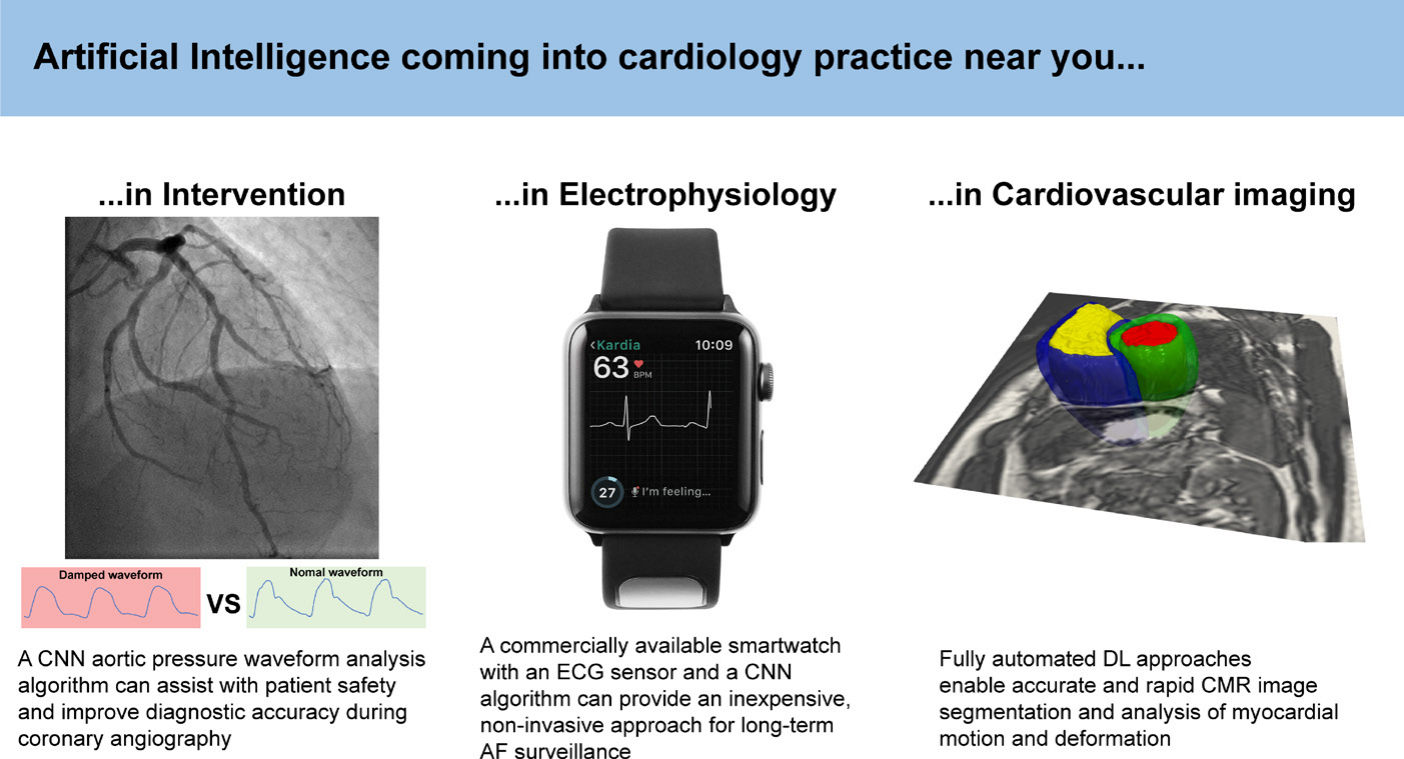
Artificial intelligence in cardiology: examples of applications in interventional cardiology,^7^ electrophysiology^5^ and imaging.^2^ AF, atrial fibrillation; CMR, cardiovascular magnetic resonance; CNN, convolutional neural network; DL, deep learning.

The use of ML in cardiovascular research has expanded exponentially in recent years, and a few pioneering applications are already being used in mainstream clinical practice. Figure 2 illustrates some recent examples, using diverse input data at different scales, in several cardiology subspecialities. Cardiovascular imaging is perhaps the area in which ML methods have been most extensively tested and have demonstrated the greatest immediate potential.^1^ These algorithms have been used for more efficient image acquisition and reconstruction, automated quality control, image segmentation, myocardial motion and blood flow analysis, and computer-assisted diagnosis. Although the use of DL approaches for complex interpretative tasks such as survival prediction from cardiac motion analysis is being evaluated,^2^ the accuracy of commercially available algorithms for routine image analysis can already match expert-level performance on real-world data.^3^

The ML interpretation of ECGs is already prevalent, and future applications are likely to assist physicians during invasive electrophysiology procedures, such as to make predictions from contact intracardiac electrograms or to predict the spatiotemporal patterns of activation in the myocardium.^4^ One of the strengths of DL approaches is the ability to tackle the challenges of scalability and high dimensionality that come with studying population-sized datasets. One such algorithm was used with a commercially available watch and an ECG sensor to carry out long-term monitoring of atrial fibrillation. This inexpensive, non-invasive approach compared well with an implantable loop recorder arm and has potential implications for population screening.^5^

Interventional cardiology has also embraced the opportunities of ML, with pilot research applications in the identification and evaluation of coronary disease from angiograms, analysis of intravascular ultrasound, non-invasive functional assessment of coronary stenosis and interpretation of pressure-wire pull back data.^6^ A specific example of such an application is the use of a neural network to perform automated pressure waveform analysis and to allow real-time identification of the characteristic ‘damping’ waveform that occurs during deep intubation of the coronary arteries.^7^ This can assist physicians in performing safer angiography, in addition to improving the diagnostic accuracy of physiological assessment of coronary stenosis.

Despite its potential, the challenges involved in the deployment of ML in frontline healthcare should not be underestimated.^8^ The generalisability of algorithms developed using high-quality data in standardised research environments to diverse real-world populations has to be thoroughly validated. Biases in the training data, model overfitting, inadequate statistical correction for multiple testing and limited transparency around the processes by which DL algorithms reach their output (‘black box’ systems) are only some of the pitfalls of AI that can have significant implications for patients and require careful evaluation by researchers, clinicians and regulatory entities.

Trainees and cardiologists interested in AI have the benefit of growing intersectoral support for the health data science revolution across the NHS, academia and industry. Leading this endeavour is Health Data Research UK (HDRUK), which is an independent organisation bringing together funders and research institutions to accelerate advanced healthcare analytics and sharing of best practice. The British Heart Foundation has also recently announced a partnership with HDRUK to enable responsible research combining the power of advanced analytical methods with the UK’s large-scale and diverse cardiovascular data resources. These and other initiatives provide an opportunity for democratising access to high-quality clinical data at scale, enabling rapid progress from proof-of-concept prototypes to real-world technology for delivering better care and accelerating biomedical research.
